# The des-Arg^9^-bradykinin/B1R axis: Hepatic damage in COVID-19

**DOI:** 10.3389/fphys.2022.1080837

**Published:** 2022-12-19

**Authors:** Gabriel Moreira de M Mendes, Israel Júnior Borges Do Nascimento, Paulo HS. Marazzi-Diniz, Izabela B. Da Silveira, Matheus F. Itaborahy, Luiz E. Viana, Filipe A. Silva, Monique F Santana, Rebecca AA. Pinto, Bruna G. Dutra, Marcus Vinicius G. Lacerda, Stanley A. Araujo, David Wanderley, Paula VT. Vidigal, Paulo HC Diniz, Thiago Verano-Braga, Robson AS. Santos, M Fatima Leite

**Affiliations:** ^1^ Departamento de Fisiologia e Biofísica, Instituto de Ciências Biológicas, Universidade Federal de Minas Gerais, Belo Horizonte, Brazil; ^2^ Faculdade de Farmácia, Universidade Federal de Minas Gerais, Belo Horizonte, Brazil; ^3^ Escola de Medicina e Hospital universitário, Universidade Federal de Minas Gerais, Belo Horizonte, Brazil; ^4^ Center for Infectious Disease Research, Medical College of Wisconsin, Milwaukee, WI, United States; ^5^ Departamento de Anatomia Patológica e Medicina Legal, Escola de Medicina, Universidade Federal de Minas Gerais, Belo Horizonte, Brazil; ^6^ Universidade do Estado do Amazonas, Manaus, Brazil; ^7^ Universidade Federal do Amazonas, Manaus, Brazil; ^8^ Instituto Mineiro de Nefropatologia, Belo Horizonte, Brazil; ^9^ Departamento de Clínica Médica, Escola de Medicina, Universidade Federal de Minas Gerais, Belo Horizonte, Minas Gerais, Brazil

**Keywords:** SARS-cov-2, COVID-19, hepatic damage, contact system, bradykinin, bradykinin 1 receptor, kinin-kallikrein system, renin-angiotensin system

## Abstract

Patients infected by the SARS-CoV-2 virus are commonly diagnosed with threatening liver conditions associated with drug-induced therapies and systemic viral action. RNA-Seq data from cells in bronchoalveolar lavage fluid from COVID-19 patients have pointed out dysregulation of kallikrein-kinin and renin-angiotensin systems as a possible mechanism that triggers multi-organ damage away from the leading site of virus infection. Therefore, we measured the plasma concentration of biologically active peptides from the kallikrein-kinin system, bradykinin and des-Arg^9^-bradykinin, and liver expression of its proinflammatory axis, bradykinin 1 receptor (B1R). We measured the plasma concentration of bradykinin and des-Arg^9^-bradykinin of 20 virologically confirmed COVID-19 patients using a liquid chromatography-tandem mass spectrometry-based methodology. The expression of B1R was evaluated by immunohistochemistry from post-mortem liver specimens of 27 COVID-19 individuals. We found a significantly higher blood level of des-Arg^9^-bradykinin and a lower bradykinin concentration in patients with COVID-19 compared to a healthy, uninfected control group. We also observed increased B1R expression levels in hepatic tissues of patients with COVID-19 under all hepatic injuries analyzed (liver congestion, portal vein dilation, steatosis, and ischemic necrosis). Our data indicate that des-Arg^9^-bradykinin/B1R is associated with the acute hepatic dysfunction induced by the SARS-CoV-2 virus infection in the pathogenesis of COVID-19.

## Introduction

Since the beginning of the ongoing severe acute respiratory syndrome coronavirus 2 (SARS-CoV-2) pandemic, many reports have suggested direct and indirect viral-mediated damage in multiple organs, including the liver (Bertolini et al., 2020; Zarifian et al., 2020). Epidemiologically, approximately 22% of patients who evolved with COVID-19, a critical illness caused by SARS-CoV-2, develop an acute liver injury (Nascimento et al., 2020a; Nascimento et al., 2020b). Some factors contribute to this high prevalence of critical hepatic damage. Firstly, pre-existing chronic liver diseases strongly correlate with morbidity and mortality (Ahmad et al., 2021; Liu et al., 2022). However, this theory was refuted as evidence-based studies showed no worse prognosis among patients with previous liver pathologies (Guan et al., 2020; Kulkarni et al., 2020). Furthermore, acute liver failure could be caused by drug-induced liver injury, as COVID-19 patients are exposed to extensive pharmacological therapies, and most drugs are metabolized in the liver (Guan et al., 2020; Ortiz et al., 2021; Sodeifian et al., 2021). Interestingly, the likelihood of absolute *in-situ* viral-induced liver damage has not been proved yet, as SARS-CoV-2 is not often detected in liver *post-mortem* specimens (Duarte-Neto et al., 2021; Santana et al., 2021). Therefore, the acute hepatic injury observed during the natural course of COVID-19 might reflect a potential systemic complication caused by the primarily SARS-CoV-2 lung infection (Lindner et al., 2020; Chippa et al., 2022).

Several studies have evidenced the effect and potential roles of components of the renin-angiotensin and the kallikrein-kinin systems in injuries observed in SARS-CoV2 infected patients (Meini et al., 2020; Pucci et al., 2021). The angiotensin-converting enzyme 2 (ACE2) mediates the SARS-CoV-2 entrance into the cell and cleavages des-Arg^9^-bradykinin (des-Arg^9^-BK) into inactive metabolites, and the conversion of angiotensin I and angiotensin II to the non-apeptide angiotensin 1-9 and heptapeptide angiotensin 1-7, respectively (Sidarta-Oliveira et al., 2020; Sugawara et al., 2021; Tabassum et al., 2022). Once formed, des-Arg^9^-BK binds to bradykinin 1 receptor (B1R), increasing vascular permeability, neutrophil recruitment, and prompting broncho- and vasoconstriction and inflammation (Sodhi et al., 2018; Van de Veerdonk et al., 2020). In addition, studies have described the likely occurrence of the “*bradykinin-related peptides storm*” at the pulmonary site in addition to events related to the “*cytokine storm*” during the clinical progress of COVID-19 (Karamyan, 2021; Yin et al., 2021), which altogether could positively modulate the expression of the B1R in the lung as well as in organs distant from the SARS-CoV-2 infection site (Zamorano Cuervo and Grandvaux, 2020; McCarthy et al., 2021). Therefore, our study aimed to evaluate whether SARS-CoV-2 infection alters the circulating levels of des-Arg^9^-BK and liver expression of its receptor, B1R.

## Material and methods

Experimental design: We conducted the biochemical assessment using peripheral venous blood from healthy, controlled individuals that do not take ACE inhibitor medication and SARS-CoV-2-infected individuals. In addition, the histopathological assessment was performed using liver specimens from colon cancer patients (control group) and *post-mortem* liver from SARS-CoV-2-infected individuals. Control group patients assigned to the histopathological assessment deceased from non-hepatic-related causes (including myocardial infarction, renal failure, and respiratory distress), while those evaluated in the experimental group deceased due to COVID-19-associated complications.

This study was approved by the local ethical board (CAAE 30152620.1.0000.0005) and was executed in strict accordance with the directive declaration of Helsinki for medical research involving humans. The patients in the experimental group were admitted to a designated hospital between March 23 and 18 May 2020, underwent initial clinical evaluation by a trained physician, and tested positive for SARS-CoV-2 confirmed by quantitative real-time polymerase chain reaction (q-RT-PCR). Legal authorization from the next of kin of the deceased part was not required.

### Blood draw and plasma sample preparation

Approximately 2 ml of human EDTA-anticoagulated venous blood (*n* = 20–12 male and 8 female, and *n* = 23–12 male and 11 female, for SARS-CoV-2-infected individuals and healthy controlled individuals that do not take ACE inhibitor medication, respectively, both in a 56–64 age range) was draw into a vacutainer tube containing four protease inhibitors (1 mmol.L^−1^ phydroxymercuribenzoate, 30 mmol.L^−1^ 1,10-phenanthroline, 1 mmol.L^−1^ phenylmethylsulfonyl fluoride, and 1 mmol.L^−1^ pepstatin, same inhibitor cocktail used by our group in Paquette et al., 2018. Most blood draws were performed between 17–30 days after qRT-PCR for SARS-CoV-2, from hospitalized patients. Both COVID-19 and control individuals were not fully vaccinated at the moment of blood draw. Blood samples were homogenized by gentle inversion, immediately centrifuged for plasma separation (at 5,000 x rotations per minute [RPM] for 15 min, at 4°C), and stored at −80 °C until extraction. Solid-phase extraction was carried out on “Vacuum” Sep-Pak C183cc Vac RC Cartridge, containing 500 mg of sorbent (Waters, Milford, MA, United States). Two sequential washes activated the C18 resin with acetonitrile (99%, 10 ml) and trifluoroacetic acid (0.1%, 10 ml). Subsequently, the resin was rewashed with 10 ml of trifluoroacetic acid (0.1%). Afterwards, the resin was conditioned with trifluoroacetic acid (0.1%, 3 ml), bovine serum albumin (0.1%, 3 ml), and acetonitrile (10%, 10 ml). The samples were then loaded and washed with trifluoroacetic acid (0.1%, 20 ml), followed by acetonitrile (20%, 3 ml) and trifluoroacetic acid (0.1%, 3 ml). Elution was performed with trifluoroacetic acid (0.1%, 3 ml) and acetonitrile (90%, 3 ml) into low-binding polypropylene tubes (Eppendorf, Hamburg, Germany). Samples were dried using a vacuum concentrator (SpeedVac SRF110, Eppendorf, Hamburg, Germany). The dried samples were resuspended in 50 µl of 0.1% formic acid (sample concentration factor is 40).

### Liquid chromatography-tandem mass spectrometry

The method employed to quantify the kinin peptides was similar to the one used by Souza-Silva et al. (2022). The liquid chromatography-tandem mass spectrometry instrument consisted of an ACQUITY I-class UPLC system (Waters, Milford, MA, United States) and Xevo TQ-S triple quadruple mass spectrometer (Waters, Milford, MA, United States of America). The chromatographic separation was carried out in a C18 column (ACQUITY UPLC BEH C18 Column, 130 Å, 1.7 μm, 2.1 mm × 100 mm, manufactured by Waters, Milford, MA, United States) for 5.5 min, each injection (10 µl final volume per sample). Solvent A was made of 0.1% formic acid in H_2_O and solvent B of 0.1% formic acid in acetonitrile. The chromatographic gradient was set as previously described in a 300 µl.L^−1^ flow rate as follows (expressed as % of solvent B): i) 3%–40% in 3.5 min, ii) 40%–99% in 0.01 min, iii) 99% for 0.99 min, iv) 99%–3% in 0.01 min, and v) 3% for 0.99 min (Souza-Silva et al., 2022). As far as the mass spectrometry analysis is concerned, the main parameters were as follows: i) capillary = 3.5 kV; ii) cone = 20V; iii) temperature of the desolvation gas (hydrogen) = 550°C. The collision energy (CE) (argon gas) was tuned for each target peptide spanning from 10 to 20 CE. Mass spectrometry in the multiple reaction monitoring (MRM) mode were utilized to track the following transition: bradykinin (1–9): 354.4 > 419.3 and 354.4 > 408.2; des-Arg^9^-BK: 453 > 263.1 and 453 > 642.8. The calibration curve was prepared using a stock solution containing bradykinin and des-Arg^9^-BK ranging from 50 to 1000 *p*g ml^−1^. We applied the calibration curve model y = ax + b that yielded a correlation coefficient of *R* ^2^ > 0.99. The sensitivity of the assay was in the low picogram range. The low limit of quantitation (LLOQ) for bradykinin and des-Arg^9^-BK was 3.1 *p*g ml^−1^. We executed data acquisition through the software MassLynx MS (Waters, Milford, MA, United States of America). In addition, raw data was evaluated using the TargetLynx™ (Waters, Milford, MA, United States).

### Selection and preparation of tissue

We used fresh liver tissues from 27 post-mortem autopsies of COVID-19 patients who were hospitalized between March-May 2020, when COVID-19 vaccines were not available in the world yet. Clinical information of patients such as age, gender, body mass index (BMI), comorbidities before COVID-19, medication history, serum liver function tests (ALT, AST, and total bilirubin), and creatinine have been evaluated and discussed in a previous publication by our group (Santana et al., 2021). Both liver and lung specimens came from deceased COVID-19 patients, which showed SARS-CoV-2 staining in the lung, but not in the liver. Following the death, corpses were stored under low temperatures until the specimens sampling. Liver samples of 1 cm^3^ were from 4 different liver sections during the procedure and subsequently fixed in 4% formalin, preventing aerosol production. Samples were dehydrated with increasing concentrations of ethanol and impregnated with paraffin wax. Later, the paraffin compartments were sliced into 4 µm-thick sections on a semi-motorized rotatory microtome and stained with the hematoxylin and eosin (H&E) method. Two board-certified liver pathologists carefully evaluated each histological item and rated it according to the presence of steatosis, congestion, portal vein dilatation, and ischemic necrosis under blinded conditions, as previous published by our group (Santana et al., 2021). We defined ischemic necrosis as extensive centrilobular necrosis. The liver tissue from the control group was obtained from colon cancer, SARS-CoV-2-uninfected patients without clinical and laboratory signs of chronic liver disease.

### Immunohistochemistry protocol

We evaluated the expression of B1R, in hepatic tissue using immunohistochemistry (IHC) standard techniques. Furthermore, IHC was performed in the lung specimens from COVID-19 as positive control, given that it is the direct site of viral infection. Human hepatic and pulmonary tissue sections were formalin-fixed and paraffin-embedded and then de-waxed. Afterwards, antigen retrieval was performed in citrate buffer (10 mM), containing 0.6% hydrogen peroxide. Novolink Polymer Detection System (Leica Biosystems, Germany) was used, consecutively, as described in previously (Fonseca et al., 2018). In the liver tissue sections, primary Sigma-Aldrich anti-B1R (MFCD06798426) (1:200), was incubated at room temperature, overnight. Subsequently, the liver sections were incubated with detection polymer at room temperature, for 40 min. Furthermore, DAB was used for signal detection. Meanwhile, in the lung specimens we used Sigma-Aldrich anti-B1R (MFCD06798426) (1:200), followed by previously explained steps.

### Image collection and tissue analysis

We captured ten images (at ×400 magnification) per immunohistochemistry slide for each individual from different selected areas with a Leica DM2500 LED optical microscope (Life Science, Wetzlar, Germany). B1R expression was quantified using the ImageJ bundled with 64-bit Java 1.8.0 172 software (National Institute of Technology, Bethesda, MD, United States of America). Using the “freehand selection” tool, ten zones of each IHC image were randomly selected in areas containing hepatocytes (avoiding areas with bile ducts, portal vein, central vein, that are not the focus of the current work), color-inverted, and processed with the “histogram” function. Then, we collected the “histogram mean value” for statistical analyses.

### Statistics

Unless evidenced, our findings are expressed as mean values ± Standard Deviation (SD). All statistical assessments were performed in Prism (GraphPad Software, San Diego, CA). The data normality distribution was verified by Anderson-Darling test and outliers was verified by ROUT method, with Q = 1%. Differences between groups were determined using a Mann-Whitney test for non-Gaussian distribution or one-way ANOVA, followed by the Kruskal–Wallis’ test. The value of *p* < 0.05 (*), *p* < 0.01 (**), *p* < 0.001 (***) and *p* < 0.0001 (****) were statistically significant. For the plasma peptides measurement analysis, outliers and values under LLOQ were removed from the SARS-CoV-2-infected patients’ group and healthy controlled individuals.

## Results

### Bradykinin and des-Arg^9^-bradykinin concentration in human plasma of COVID-19 patients

**FIGURE 1:**
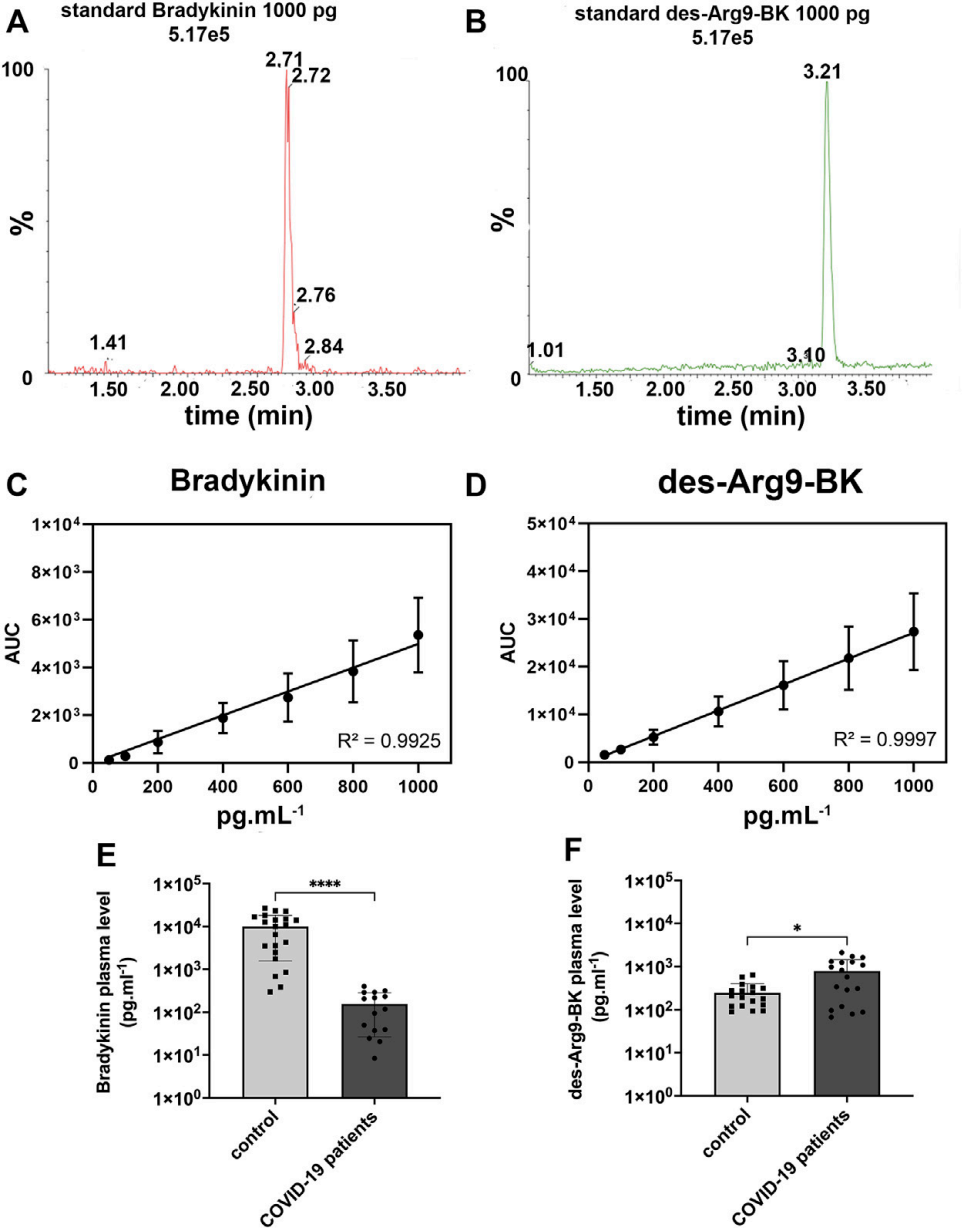
Bradykinin and des-Arg^9^-BK concentration in plasma of COVID-19 and control patients. **(A)** 1000 *p*g ml^−1^ standard chromatogram for bradykinin (BK), expressed in the graph as relative peak abundance (%) by time. **(B)** 1000 *p*g ml^−1^ standard chromatogram for des-Arg^9^-BK, expressed in the graph as relative peak abundance (%) by time. **(C)**. Calibration curve for Bradykinin and **(D)**. des-Arg^9^-BK showed a good linearity with *R* ^2^ > 0.99, **(E)**. Bradykinin concentration in human plasma was measured by LC-MS/MS from 15 COVID-19 patients (156.4 ± 129.8 *p*g ml^−1^) and compared with 21 control patients (9,929 ± 8,368 *p*g ml^−1^). Concentration of bradykinin was lower in the plasma of COVID-19 patients (****p < 0.0001). *Y*-axis is in logarithmic scale. **(F)**. Des-Arg^9^-BK concentration in human plasma was measured by LC-MS/MS from 18 COVID-19 patients (786.3 ± 658.4 *p*g ml^−1^) and compared with 18 control patients (246.0 ± 157.5 *p*g ml^−1^). The concentration of des-Arg^9^-BK was higher in the plasma of COVID-19 patients (*p < 0.05). *Y*-axis is in logarithmic scale.

The retention time of each kinin was 2.70 (bradykinin) and 3.20 min (des-Arg^9^-BK), as shown in Figure 1A and Figure 1B Respectively. Our LC-MRM method yielded a good linearity as seen by the high correlation coefficient (Figures 1C, D). As far as the bradykinin mean concentration is concerned, we observed a statistically significant lower concentration (*p < 0.0001*) of bradykinin among 15 patients in the COVID-19 group (156.4 ± 129.8 *p*g ml^−1^ *)* than in the 21 healthy individuals on control group (9,929 ± 8,368 *p*g ml^−1^). COVID-19 patients’ bradykinin measurements are available in Figure 1E. With regards to the des-Arg^9^-BK plasma concentrations, we verified a significant higher (*p < 0.05*) mean concentration among 18 patients in COVID-19 group (786.3 ± 658.4 *p*g ml^−1^) than in 18 volunteers on the control group (246.0 ± 157.5 *p*g ml^−1^). COVID-19 patients’ des-Arg^9^-BK measurements are available in Figure 1F. We found that bradykinin *versus* des-Arg^9^-BK levels is inversely correlated in 70% and 82% of the individuals in the COVID-19 group and the control group, respectively (Supplemental Table S1). These results show that des-Arg^9^-BK, a metabolite of bradykinin, increases in the plasma of SARS-CoV-2 infected patients.

### SARS-CoV-2 infection triggers expression of B1R in hepatic cells

**FIGURE 2:**
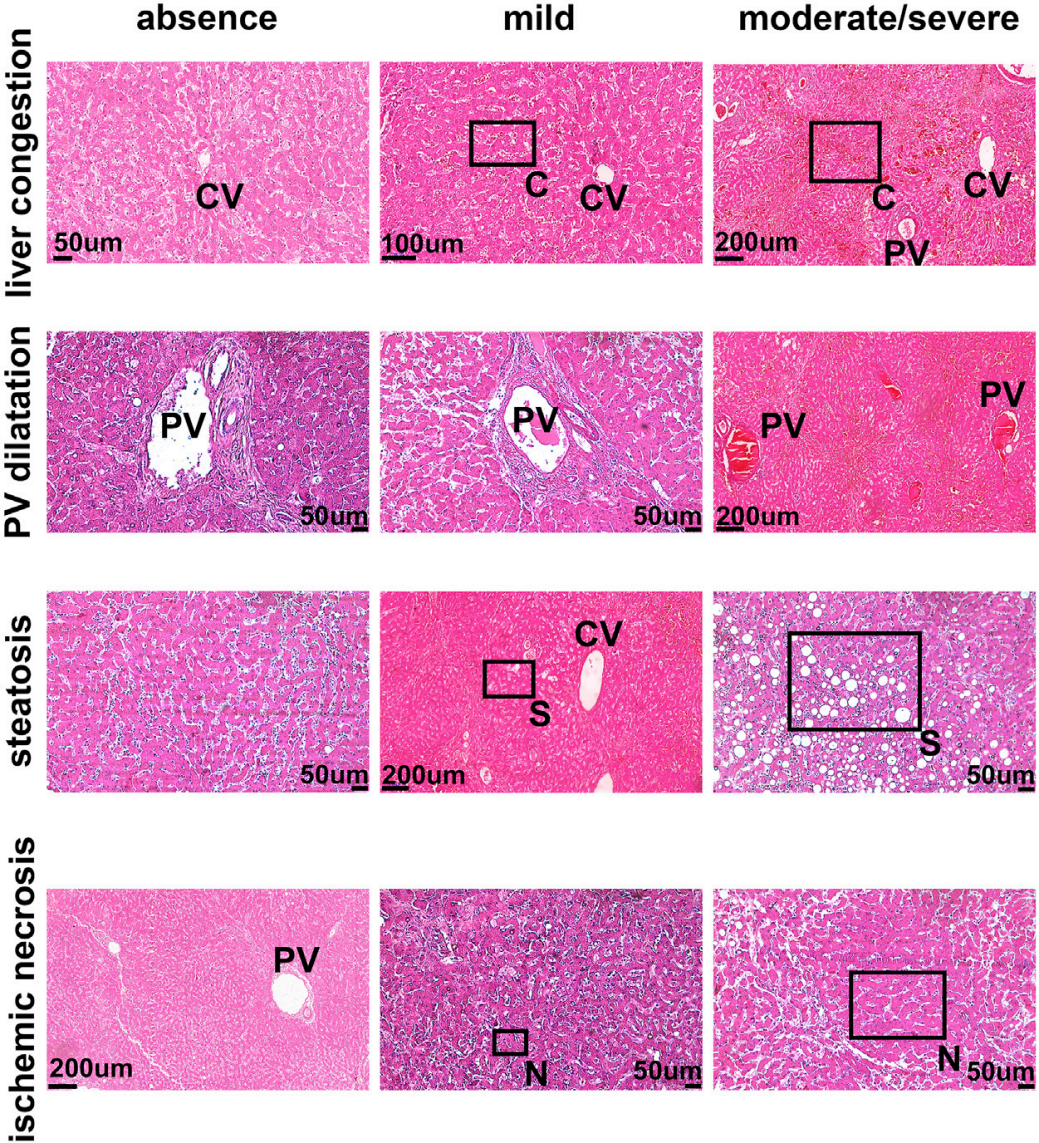
The pattern of liver damage in COVID-19 patients. The pattern of liver damage in hematoxylin and eosin staining images is representative of absent, mild, and moderate/severe. PV–Portal Vein; CV–Central Vein; C–Congestion; S–Steatosis N–Necrosis. Demarcated areas highlighted in some panels indicate congestion, steatosis and ischemic necrosis.

**FIGURE 3:**
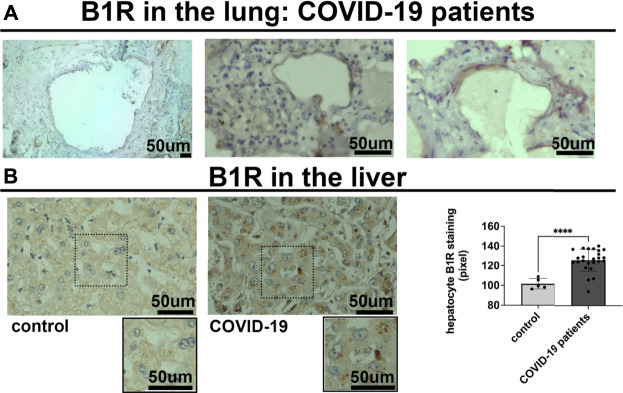
Bradykinin 1 receptor (B1R) is expressed in the lung and the liver of SARS-CoV-2 infected patients. **(A).** Representative images of immunohistochemistry (IHC) staining for B1R in the lung of 3 different COVID-19 patients, as positive control. B1R is observed in alveolar pneumocytes. **(B).** IHC staining for B1R in the liver of 25 COVID-19 patients (125.9 ± 11.0 a. u.) (on the left) and 5 SARS-CoV-2-uninfected control patients (101.6 ± 5.2 a. u.) (on the right). B1R is observed in hepatocytes. Graphs show quantification of staining intensity in arbitrary units (a.u.). B1R staining was higher in the liver of COVID-19 patients (****p < 0.0001). Scale bar = 50 µm.

**FIGURE 4:**
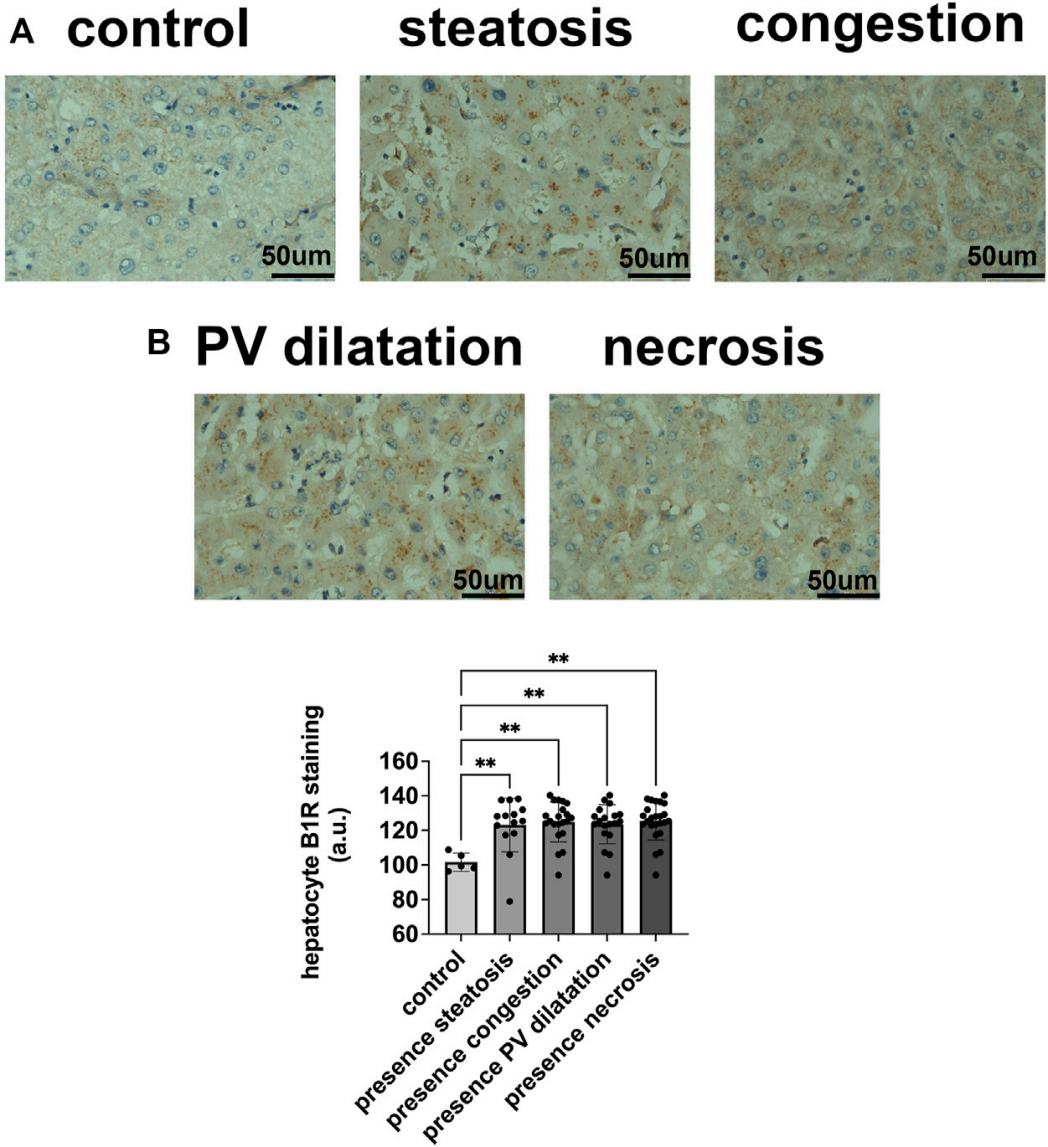
B1R expression in hepatocytes in COVID-19 patients and its correlation with patterns of liver injuries. **(A,B)**. B1R in the liver of control individuals (*n* = 5) and COVID-19 patients (*n* = 25); Comparison between B1R expression level in the liver of 5 control individuals (101.6 ± 5.2 a. u.) and liver of 14 COVID-19 patients with steatosis (123.2 ± 15.5 a. u), 21 with congestion (124.9 ± 11.5 a. u), 19 with PV dilation (123.5 ± 11.3 a. u), and 24 with ischemic necrosis (125.4 ± 11.0 a. u), (*p* < 0.01). Scale bar = 50 µm.

To determine the potential relationship between the B1R expression level and liver injuries observed in COVID-19 patients, we first evaluated the categorical variables of liver damage by hematoxylin and eosin staining. Categorical variables regarding the presence or absence of four major liver injuries (portal vein dilatation, ischemic necrosis, steatosis, and congestion) are shown in Figure 2. It is well known that inflammation is a primary stimulus that induces tissue expression of B1R (Ahluwalia and Perretti, 1999; Vellani et al., 2004). Even though the lung is the main target of SARS-CoV-2 infection and tissue damage, liver injuries are often observed in COVID-19 patients. Therefore, we investigated whether the expression of B1R is altered in patients infected with SARS-CoV-2, which is essentially driven by the increased inflammatory status during the natural history of the disease. Pulmonary staining was used as a positive control since it is the leading site of SARS-CoV-2 infection (Figure 3A). We found that B1R expression in the hepatic tissues of COVID-19 patients was significantly greater than in the control group (*p < 0.0001*), as shown Figure 3B. In the liver, the mean B1R expression level among the COVID-19 group (*n* = 25) was 125.9 ± 11.0 a. u., while in the control group, it was 101.6 ± 5.2 a. u. Overall, the B1R expression reflected histological and functional liver injury (Figure 4). Our findings show that the presence of steatosis, congestion, portal vein dilation and ischemic necrosis correlated with an increased expression of B1R (*p < 0.01*). Registered mean score expression (MSE) among COVID-19 individuals were 123.2 ± 15.5 a. u. For steatosis, 124.9 ± 15.5 a. u. For congestion, 123.5 ± 11.3 a. u. For portal vein dilation, 125.4 ± 11.0 a. u for ischemic necrosis and 101.6 ± 5.3 a. u. For control individuals. Together, these findings show an association between increased B1R expression level and the presence of hepatic injuries in the liver of patients infected with SARS-CoV-2.

## Discussion

Hepatic dysfunction, commonly reported in COVID-19 patients, is associated with poor clinical outcomes and appears of multifactorial origin (Kulkarni et al., 2020; Sodeifian et al., 2021). SARS-CoV-2 viral inclusions have not often been identified in the liver tissue of adult patients (Santana et al., 2021), which indicates additional inputs for the observed hepatic damage in COVID-19. It has been shown that ACE2 is not the only pathway for SARS-CoV-2 entry into target cells. For instance, TMPRSS2 is required to cleave the S domain to initiate the viral-ACE2 internalization. Therefore, just ACE2 increase alone is not the only explanation for tissue virus infection, corroborating our previously published finding that although ACE2 is increased in the liver of COVID-19 patients, no SARS-CoV-2 was found in the identical specimens (Santana et al., 2021). Moreover, cytokine storms can occur in severe SARS-CoV-2 infection (Mangalmurti and Hunter, 2020), and it is higher in subjects with liver dysfunction than those with normal hepatic tests (Premkumar and Kedarisetty, 2021; Taneva et al., 2021). Besides the pro-inflammatory role (Fara et al., 2020), cytokines regulate the expression level of several molecules, including the bradykinin receptors (Ricciardolo et al., 2018). Therefore, understanding the biological dynamic of bradykinin and its metabolite des-Arg^9^-BK, as well as assessing the expression of correlated receptors in the liver, is of utmost importance since the kinin-kallikrein system activation opposes the effects of the renin-angiotensin system, setting up an essential counter-control mechanism, that can either deflagrate protective or harmful regulatory axes depending on the prevailing signaling pathway.

Based on previous studies demonstrating that SARS-CoV-2 binds to the ACE2 enzyme (Jackson et al., 2021), it is possible to postulate that SARS-CoV-2/ACE2 binding might impairs the inactivation process of des-Arg^9^-BK, as ACE2 is well-known to degrade des-Arg^9^-BK (Sodhi et al., 2018; Tabassum et al., 2022). In this aspect, it has been suggested that kidney injury caused by SARS-CoV-2 could also be due, at least in part, to the exacerbation of des-Arg^9^-BK/B1R axis effects (Azinheira Nobrega Cruz et al., 2021), as it has been shown here. Thus, less des-Arg^9^-BK inactivation combined with its longer systemic blood half-life (Cyr et al., 2001; Jackson et al., 2021) might partially explain the higher circulating levels of des-Arg^9^-BK in COVID-19 patients. Moreover, it has been demonstrated that the expression of ACE1, which cleaves bradykinin into the metabolites bradykinin1-7 but not bradykinin1-5, as shown recently (Souza-Silva et al., 2022), is higher in severe COVID-19 patients (Akbari et al., 2022), representing one way by which bradykinin level is decreased in the plasma of SARS-CoV-2 infected individuals in comparison with the control group. Therefore, evaluating bradykinin1-7 and bradykinin1-5 would be of great importance to better elucidate the kinin pathways in COVID-19 pathology, as suggested by Souza-Silva et al. (2022). Additionally, it was recently reported that carboxypeptidase N subunit 1, which cleaves bradykinin into des-Arg^9^-BK, is overexpressed in COVID-19 patients (Alfaro et al., 2022). This can result in lower bradykinin and overaccumulation of circulating des-Arg^9^-BK, as observed in COVID-19 patients in this current study. A lower bradykinin level might reduce the beneficial systemic effect of the bradykinin/B2 receptor axis. It is important to consider that the actual human plasma concentration of bradykinin and des-Arg^9^-BK can vary largely depending on the protease inhibitor cocktail used for blood draw as well as differences in the liquid chromatography-tandem mass spectrometry assay (Lindström et al., 2019; Gangnus and Burckhardt, 2021a). In the current study, we used a cocktail of protease inhibitors without a selective kallikrein inhibitor. The cocktail prevented bradykinin degradation, but it did not prevent kallikrein to generate bradykinin, altogether representing one of the reasons why plasma bradykinin level reported in the current work is higher than mentioned in other studies. Despite these variations, we demonstrate that the amount of bradykinin and des-Arg^9^-BK is significantly different between control and COVID-19 patients. An increase in plasma levels of angiotensin 1–7 has been observed in SARS-CoV-2-infected individuals (Martins et al., 2021; Miesbach, 2020, suggesting that the Angiotensin 1–7/MASR axis could have a counter-control effect in COVID-19 pathogenesis. However, the anti-inflammatory, anti-fibrotic, anti-thrombotic, and vasodilatory impacts would not be entirely achieved because the activation of MASR is not equally functional in veins compared to other vascular beds, such as arteries, capillaries, and microcirculation sites (Feterik et al., 2000; Souza dos Santos et al., 2001). Furthermore, most liver histopathological alterations observed in COVID-19 patients (including ischemic necrosis and congestion) are present in conditions that lead to acute liver injuries, such as cardiogenic shock, lung hypertension, and *cor pulmonale*, common lethal mechanisms observed in patients with severe COVID-19.

Besides the cytokine storm, systemic manifestations of SARS-CoV-2 lung infection have also been attributed to alterations of the bradykinin storm (Garvin et al., 2020; Gangnus & Burckhardt, 2021b). Bradykinin storm can cause vasodilation, vascular permeability, and hypotension, leading to severe clinical symptoms such as lung edema, cardiovascular dysfunction, and thromboembolism (Ahmad et al., 2021). The misregulation of both renin-angiotensin and kinin-kallikrein systems can also explain many liver injuries in COVID-19 patients, such as hepatic vascular resistance and sinusoidal capillarization, portal hypertension, and edema formation. ACE2/Ang-(1–9)/AT2R and ACE2/Ang-(1–7)/MASR alternative renin-angiotensin system axes are associated with promoting vasodilation, proapoptotic and anti-inflammatory effects, reducing hepatic fibrogenesis and portal hypertension (Shim et al., 2018). In contrast, des-Arg^9^-BK is related to immune liver injury. However, the mechanism of B1R meditated immune dysfunction is not well clarified (Zhang et al., 2018). It is known that B1R is minimally expressed in healthy tissues (Leeb-Lundberg et al.*,* 2005) and that inflammation induces B1R expression (Ahluwalia and Perretti, 1999; Vellani et al., 2004). Since COVID-19 is associated with hyperinflammatory response, expression of B1R in the liver might be a result of the systemic inflammation caused by SARS-CoV-2 infection. Indeed, we found that liver specimens from COVID-19 patients showed an overexpression of B1R even higher than the control liver specimens from colon cancer patients. In this context, the build-up of circulating blood levels of des-Arg^9^-BK, in the presence of increased expression of its receptor, B1R, in the liver, can trigger the pro-inflammatory axes of the kinin pathway in the related organ. Thus, the upregulation of B1R might play an essential role in eliciting intracellular signalization for liver damage. Liver damage associated with increased B1R expression has already been demonstrated as a consequence of malaria infection, and anti-malarial chloroquine treatment is also related to increased B1R signaling (Ventura et al., 2019). Therefore, we cannot rule out that the high liver B1R expression observed in COVID-19 could be triggered not just by the systemic effect of SARS-CoV-2 infection but also by hydroxychloroquine taken by some patients in the current study (Santana et al., 2021).

A limitation of the current study is that we used a restricted set of patients and the evaluation based on samples from a specific geographic area in Brazil, which ought to be interpreted with caution and conceivably translated to multiple populations. However, given the many challenges of the pandemic period, in which it is hard to get biological specimens for analysis, the number of patients could be considered relevant. Most importantly, as a strength, our study filled the literature inconsistency and knowledge gap previously stressed by several top-ranked publications regarding the kinin-kallikrein system regulation in COVID-19 patients.

Overall, the overexpression of B1R found in hepatic parenchymal cells associated with SARS-CoV-2 infection and the observed increase in circulating des-Arg^9^-BK could lead to subsequent release of additional cytokines, such as IL-6, IL-8, nitric oxide, and others, implicating in a severe inflammatory state and vascular breakdown. The activated pro-inflammatory cascade events in the natural history of COVID-19 support the establishment of an acute hepatic inflammatory condition, which might lead to loss of tissue integrity and functionality, not fully counterbalanced by possible compensatory mechanisms. Therefore, dysregulation of the kinin-kallikrein system shown in the current work could substantially lead to acute hepatic injury and increased mortality in COVID-19. The findings in our study should not be seen as an isolated factor but as one of many factors that alternatively elucidate the pathology of COVID-19-related organ injury.

## Data Availability

The original contributions presented in the study are included in the article/Supplementary Material, further inquiries can be directed to the corresponding author.
