# Supplementary material for: The des-Arg9-bradykinin/B1R axis: Hepatic damage in COVID-19
Source: Front Physiol. 2022 Dec 19;13:1080837. doi: 10.3389/fphys.2022.1080837 (PMC9806358; doi:10.3389/fphys.2022.1080837)
Supplement: Supplementary file 1 [file Table1.DOCX]

**Supplemental table 1.**

|  | **bradykinin (pg.ml^-1^)** | **des-Arg^9^-bradikynin (pg.ml^-1^)** |
| --- | --- | --- |
| **Patient 1** | 216.242798817536 | 309.124018150578 |
| **Patient 2** | 49.8381473886959 | 825.164005606458 |
| **Patient 3** | 24.5827131167222 | 3632.3836524750900 |
| **Patient 4** | 117.585691026426 | 79.0930372891743 |
| **Patient 5** | 290.542396282093 | 1630.70109946365 |
| **Patient 6** | 205.503320460667 | 56.8796153187279 |
| **Patient 7** | 1.48114142765672 | 1247.05185901842 |
| **Patient 8** | 94.4687208559815 | 1308.05724271929 |
| **Patient 9** | 316.090401700271 | 118.474394502768 |
| **Patient 10** | 1025.933445936554000 | 1286.69418604895 |
| **Patient 11** | 0.0461954441374414 | 95.9102021160176 |
| **Patient 12** | 292.027387330095 | 573.418354460289 |
| **Patient 13** | 2.28571207971715 | 66.9697455752107 |
| **Patient 14** | 39.3527439745829 | 337.294002412785 |
| **Patient 15** | 401.100605369111 | 288.588771230728 |
| **Patient 16** | 37.2325655696916 | 974.567328607359 |
| **Patient 17** | 8.35463855327352 | 1086.74387638087 |
| **Patient 18** | 20.5983560598679 | 1721.36597689885 |
| **Patient 19** | 232.928015796928 | 87.4575626010289 |
| **Patient 20** | 3439.41634970734000 | 2115.92978455859 |
|  |  |  |
| **Control 1** | 1743.51903909126 | 89.1867511376505 |
| **Control 2** | 384.05833610274 | 178.916100263021 |
| **Control 3** | 2.12980245575329 | 5097.2127157324800 |
| **Control 4** | 14017.3723361497 | 904.3133263698820 |
| **Control 5** | 688.399696510721 | 749.2125136983960 |
| **Control 6** | 297.049217069869 | 6379.6643504601900 |
| **Control 7** | 14983.8002756067 | 250.741191390347 |
| **Control 8** | 2.39927587988837 | 1181.5657205285100 |
| **Control 9** | 852.765802908023 | 94.6422371692183 |
| **Control 10** | 6513.81346729899 | 123.178481455342 |
| **Control 11** | 3590.81063242716 | 120.658116081049 |
| **Control 12** | 14141.1440821533 | 568.89904622126 |
| **Control 13** | 3529.13888177984 | 158.174183129501 |
| **Control 14** | 4271.61340575243 | 129.392870904483 |
| **Control 15** | 26617.6260435198 | 315.230056235801 |
| **Control 16** | 2478.50024297421 | 93.4439049618884 |
| **Control 17** | 11058.5285968731 | 208.019334771842 |
| **Control 18** | 18125.0487243595 | 276.891427819658 |
| **Control 19** | 12591.1926769956 | 272.571563579768 |
| **Control 20** | 9938.36970002787 | 191.363050571184 |
| **Control 21** | 16691.4796352382 | 336.758357746838 |
| **Control 22** | 22834.7943215528 | 385.34782794989 |
| **Control 23** | 23168.3129507845 | 635.341465703464 |
