# Supplementary figures and images for: The des-Arg9-bradykinin/B1R axis: Hepatic damage in COVID-19
Source: Front Physiol. 2022 Dec 19;13:1080837. doi: 10.3389/fphys.2022.1080837 (PMC9806358; doi:10.3389/fphys.2022.1080837)

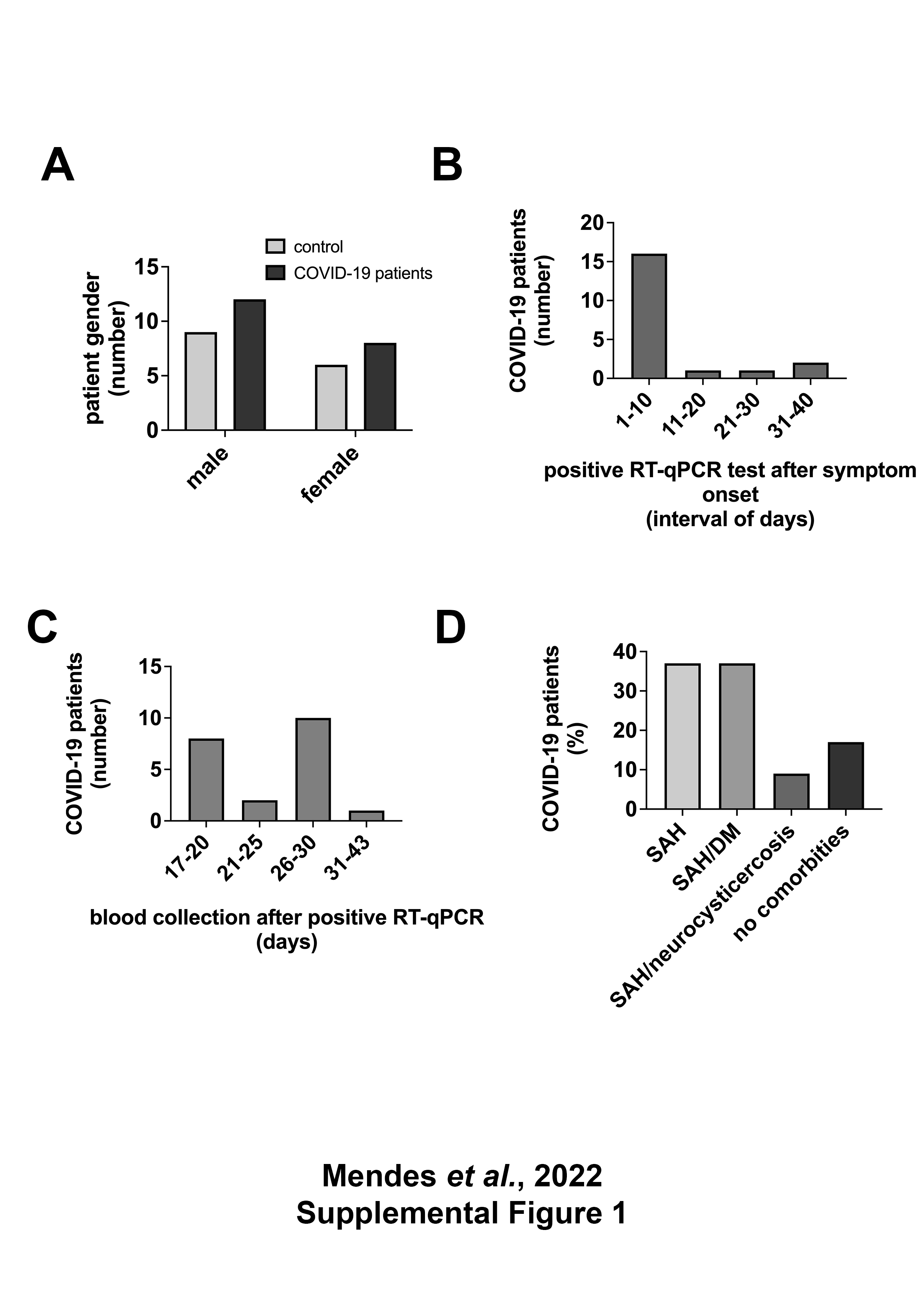

Supplement: Supplementary file 2 [file Image1.JPEG]
